# A study on interoperability between two Personal Health Train infrastructures in leukodystrophy data analysis

**DOI:** 10.1038/s41597-024-03450-6

**Published:** 2024-06-22

**Authors:** Sascha Welten, Marius de Arruda Botelho Herr, Lars Hempel, David Hieber, Peter Placzek, Michael Graf, Sven Weber, Laurenz Neumann, Maximilian Jugl, Liam Tirpitz, Karl Kindermann, Sandra Geisler, Luiz Olavo Bonino da Silva Santos, Stefan Decker, Nico Pfeifer, Oliver Kohlbacher, Toralf Kirsten

**Affiliations:** 1 RWTH Aachen University, Chair of Computer Science 5, Aachen, 52074 Germany; 2 University Hospital Tübingen, Institute for Translational Bioinformatics, Tübingen, 72072 Germany; 3 Methods in Medical Informatics, University Tübingen, Tübingen, 72076 Germany; 4 Mittweida University of Applied Sciences, Faculty Applied Computer and Bio Sciences, Mittweida, 09644 Germany; 5 Leipzig University Medical Center, Dept. Medical Data Science, Leipzig, 04107 Germany; 6 Leipzig University, Institute for Medical Informatics, Statistics and Epidemiology, Leipzig, 04107 Germany; 7 RWTH Aachen University, Data Stream Management and Analysis, Aachen, 52074 Germany; 8 Fraunhofer Institute for Applied Information Technology FIT, Sankt Augustin, 53757 Germany; 9 University of Twente - Enschede, Services and Cybersecurity Group, Faculty of Electrical Engineering, Mathematics and Computer Science, 7513 GB Enschede, the Netherlands; 10 Leipzig University, Center for Scalable Data Analytics and Artificial Intelligence, Leipzig, 04107 Germany

**Keywords:** Health care, Research data

## Abstract

The development of platforms for distributed analytics has been driven by a growing need to comply with various governance-related or legal constraints. Among these platforms, the so-called Personal Health Train (PHT) is one representative that has emerged over the recent years. However, in projects that require data from sites featuring different PHT infrastructures, institutions are facing challenges emerging from the combination of multiple PHT ecosystems, including data governance, regulatory compliance, or the modification of existing workflows. In these scenarios, the interoperability of the platforms is preferable. In this work, we introduce a conceptual framework for the technical interoperability of the PHT covering five essential requirements: Data integration, unified station identifiers, mutual metadata, aligned security protocols, and business logic. We evaluated our concept in a feasibility study that involves two distinct PHT infrastructures: PHT-meDIC and PADME. We analyzed data on leukodystrophy from patients in the University Hospitals of Tübingen and Leipzig, and patients with differential diagnoses at the University Hospital Aachen. The results of our study demonstrate the technical interoperability between these two PHT infrastructures, allowing researchers to perform analyses across the participating institutions. Our method is more space-efficient compared to the multi-homing strategy, and it shows only a minimal time overhead.

## Introduction

### Motivation and current state of the art

During the last decade, data-driven methodologies have found applications in a variety of research disciplines and have become even more essential with the introduction of more sophisticated data analyses such as Machine/Deep Learning^1,2^. Particularly in the healthcare domain, advanced analytics obtaining information from medical data has considerable potential to improve patients’ quality of life, enable more precise diagnostics and personalized treatments, and make healthcare infrastructures more efficient^3,4^. Data as such can only demonstrate its potential if it is available in a sufficiently large amount. However, due to its highly sensitive nature, patient data for research purposes is often sparse^5^. Furthermore, recently introduced data protection regulations have made accessing and moving healthcare data more challenging, e.g., due to data immanent privacy risks^6,7^. As a consequence, external access for research purposes is often limited or even prohibited, leading to data becoming siloed within individual healthcare institutions^6,8^. This means that data is typically required to remain under the control of its originating entity. This situation motivates the adoption of methods for *Distributed Analytics* (DA), such as *Federated Learning* (FL), that have been introduced to overcome these challenges of accessing and performing data analysis on distributed privacy-sensitive data^8,9^. The topic of DA has been considered and investigated in multiple initiatives and organizations, such as the European Health Data Space (EHDS - https://health.ec.europa.eu/publications/proposal-regulation-european-health-data-space_en), the German Medical Informatics Initiative (MII - https://www.medizininformatik-initiative.de/en/start), or the German National Research Data Infrastructure initiative (NFDI - https://www.nfdi.de/?lang=en). The main principle of DA results in a paradigm shift from pooling data at a single place to bringing the analysis task to the data^9^. This approach, by design, maintains the data controller’s sovereignty over the data, as it never leaves its original location. Moreover, this approach avoids moving high volumes of large medical data, e.g., Magnetic Resonance Images (MRI) or omics data, including high-throughput sequencing^7,10^.

A prominent representative of DA is the *Personal Health Train (PHT)*^11–14^. The PHT was created in the context of the GO FAIR initiative to facilitate privacy-preserving data analysis in a distributed fashion on medical data that has been collected (a) in hospitals representing a patient’s state and documenting all taken actions and (b) in medical research projects or studies, such as clinical trials (https://www.go-fair.org/implementation-networks/overview/personal-health-train/). As part of the GO FAIR PHT Implementation Network, according to its manifesto, the German National Chapter focuses on the creation of standards, guidelines, specifications, and reference implementations for the core components of the PHT^15^. Since 2019, the German National Chapter has introduced two PHT ecosystems that have been developed and implemented in conjunction with various consortia (SMITH, HiGHmed, DIFUTURE, MIRACUM) of major initiatives in Germany (https://www.medizininformatik-initiative.de/en/node/5): One is the PHT implementation by de Arruda Botelho Herr *et al*., also known as PHT-meDIC (https://personalhealthtrain.de/), and the second is the Platform for Analysis and Distributed Machine learning for Enterprises (PADME - https://padme-analytics.de/) by Welten *et al*.^16,17^. While PADME was developed in coordination with SMITH and HiGHmed, PHT-meDIC was created in coordination with DIFUTURE and MIRACUM. Both systems were designed to operate on the basis of the specific assumptions established by the respective MII projects for which they were developed: PADME places a strong emphasis on service-oriented aspects, whereas PHT-meDIC focuses more on security aspects.

Although each infrastructure has demonstrated its capabilities across multiple projects, the ongoing challenge within the PHT context is to establish interoperability between different PHT infrastructures. This shortcoming is associated with the inherent complexity and multifaceted nature of interoperability, which has been regarded as the most challenging aspect of the FAIR principles^18,19^. According to Benson *et al*. and Kouroubali *et al*., there are different types of interoperability, ranging from institutional, human, data, and technology interoperability^19,20^. Each type presents unique challenges that can make them hard to achieve^19,20^. In institutional interoperability, the variety of administrative procedures, legal standards, and organizational cultures presents significant challenges. Human interoperability, also known as process interoperability, requires coordination and shared understanding among people working within different systems and processes. Data interoperability faces hurdles due to non-standardized data formats and semantics across institutions. Finally, technological interoperability is complicated by the different technologies and platforms across systems, leading to issues with, e.g., compatibility. Enabling interoperability across various systems has been a long-standing problem and although interoperability offers multiple benefits and plays an important role in optimizing workflows, fostering data-sharing or collaboration, and reducing redundancies, there is a gap in exploring how analyses can be seamlessly exchanged between different PHT infrastructures^18,19,21–26^. Opening the boundaries of the infrastructure and enabling interoperability with external services (in our case, other PHT infrastructures) will facilitate the application of the FAIR principles and unlock the full potential of the PHT concept because researchers will have broader access to data sources for their data-intensive research.

### Objectives

Our aim is to elaborate a concept for interoperability among PHT ecosystems. Within this context, we define the term interoperability between PHT infrastructures as the ability to individually configure analysis tasks in one infrastructure but allow to include data endpoints that are (potentially uniquely) connected to a second infrastructure. This requires the transfer of analyses across infrastructures while adhering to the technical specifications and security requirements of each ecosystem. Thus, our work is primarily focused on the technical dimensions of interoperability, which leads to our first research question (RQ1):

- **RQ1:** What are the requirements to enable (technical) interoperability across PHT infrastructures?

**RQ1.1:** What specific architectural modifications or additional components are essential to enable interoperability between distinct PHT infrastructures, ensuring seamless analysis exchange?

**RQ1.2:** Can our solution enable cross-infrastructural data analysis within a real data use case?

In this study, given the variety of implementation methods for PHT, our focus is specifically on the concept of *Container Trains*^27^. This approach encapsulates the analysis code within a software container, which is then distributed across the PHT network^27^. Our goal is to maintain internal consistency within our involved infrastructures while investigating the potential and implications of interoperability in the scope of a Proof-of-Concept (PoC). In addition, we evaluate the feasibility of our implemented solution (RQ1.2) by conducting a data analysis using real patient data. By doing so, we aim to gain insights into the practicality and effectiveness of our solution in a real-world scenario and aim to derive benefits from our concept compared with the approach called ‘multi-homing’, i.e., hosting of two concurrent solutions in parallel^28^:

- **RQ2:** What are the potential benefits of our solution compared with multi-homing?

### Contributions and findings

The main contribution of this work is, to the best of our knowledge, the first study on interoperability in the context of the PHT and the corresponding challenges ahead. We introduce a conceptional framework for interoperability that comprises five integral layers: (0) harmonized data integration, (1) the establishment of unambiguous station identifiers, (2) the alignment of security protocols, (3) the definition of a metadata exchange schema, and (4) the overarching business logic responsible for train routing. These layers operate collaboratively to facilitate technical interoperability and are designed to comply with technical and security prerequisites. We demonstrate technical interoperability through a PoC that requires only minor modifications to the infrastructures involved, namely PADME and PHT-meDIC. We evaluate our solution through a real-world data use case that involves three institutions, the University Medical Center Leipzig, the University Hospitals Tübingen, and Aachen, all utilizing distinct PHT implementations within the context of the Leuko-Expert project (https://leukoexpert.hs-mittweida.de/).

We show that our PoC successfully facilitates interoperability, allowing us to exchange Container Trains between these two infrastructures and, ultimately, conduct cross-infrastructural data analyses. Furthermore, our PoC adheres to the security concepts in both infrastructures without making significant adjustments to the internal workflows (RQ1). We also demonstrate the advantages of our PoC in relation to resource efficiency and timely overhead (RQ2). Consistent with the objectives outlined in the manifesto of the German National Chapter (GO FAIR) described earlier, our work provides multiple impulses for PHT community to achieve interoperability at technical (and to some extent semantic) levels^15^. Additionally, it sets a reference for further adaptations in the interoperability of the PHT as a whole. From the perspective of end-users, particularly scientists utilizing either of these infrastructures, our findings offer considerable benefits. Users can retain their preferred infrastructure while our interoperability concept allows them to increase their datasets, thereby expanding the scope of their research studies.

The remainder of this work is structured as follows. *Results* covers the concept of our work, its corresponding PoC, and the results of our data analysis as part of our feasibility study. *Discussion* summarizes the findings from our feasibility study and discusses the interoperability aspect, along with its advantages and disadvantages. The last section *Methods* presents the two involved infrastructures and gives an overview about interoperability in general.

## Results

**Fig. 1:**
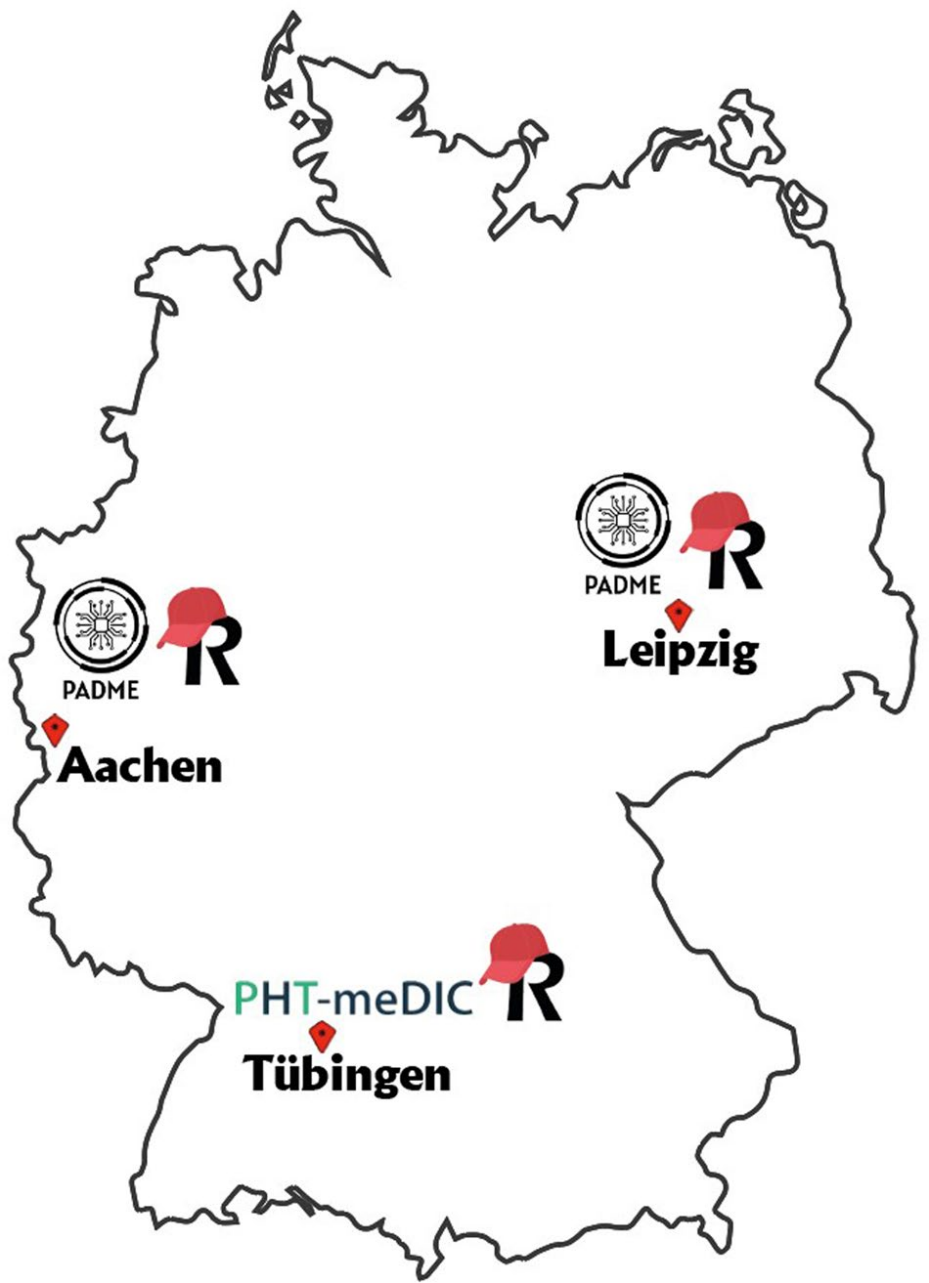
Deployment of stations within the Leuko-Expert project. Leipzig and Aachen use a PADME station, whereas Tübingen utilizes a PHT-meDIC station. In Layer 0 (see Fig. 2), a Research Electronic Data Capture (REDCap) system is implemented at each institution to facilitate data provision. Since two PHT implementations are present, their interoperability might be desirable to enlarge the global dataset.

Within this section, we present our conceptual framework for interoperability, provide an overview of the practical implementation details of our PoC, and describe the evaluation of our PoC applied in a feasibility study with real-world patient data. Our PoC is applied to a study within the Leuko-Expert project funded by the German Ministry of Health. The objective of the project is to develop an expert system to aid in the diagnosis of the rare disease (RD) leukodystrophy, a genetic disorder that affects the brain and causes movement and sensory perception disturbances^29^. The project involves clinical and genetic data that has been collected and generated by the clinics in Aachen, Leipzig, and Tübingen (see Fig. 1) in Germany. In detail, the patient data is collected from two reference centers specialized in both childhood and adult variants of leukodystrophies, located at the University Hospitals of Tübingen and Leipzig. Furthermore, data is also collected from patients who received differential diagnoses at the University Hospital Aachen. Regarding data provision, the project relies on the data integration centers (DICs - https://www.medizininformatik-initiative.de/en/consortia/data-integration-centres) at the corresponding university hospitals that are part of the MII and facilitate external access to the data. The patient data used in this study is subject to privacy and ethical considerations and, as such, is not publicly available. Access to the data is restricted to protect patient confidentiality and comply with ethical guidelines. Data access will be provided upon request, taking into account the data-sharing infrastructure. To apply for data access, we refer to the Research Data Portal for Health (https://forschen-fuer-gesundheit.de, currently available only in German) or contact the DICs directly in Aachen, Leipzig, and Tübingen.

Our designed analysis ‘Data Discovery’ encompasses the execution of a train exchanged between the infrastructures PADME and PHT-meDIC. In the Leuko-Expert scenario, the interoperability between these infrastructures to analyze more data becomes desirable, since two DICs have deployed PADME and one has deployed PHT-meDIC (see Fig. 1). For exact implementation details, see our supplementary materials^30^. Note that the purpose of the feasibility study performed is to assess the practicability of our PoC and the selected analyses are arbitrary. Further, note that when we refer to interoperability, we are using it synonymously with technical interoperability or horizontal interoperability.

### Conceptual framework for interoperability

**Fig. 2:**
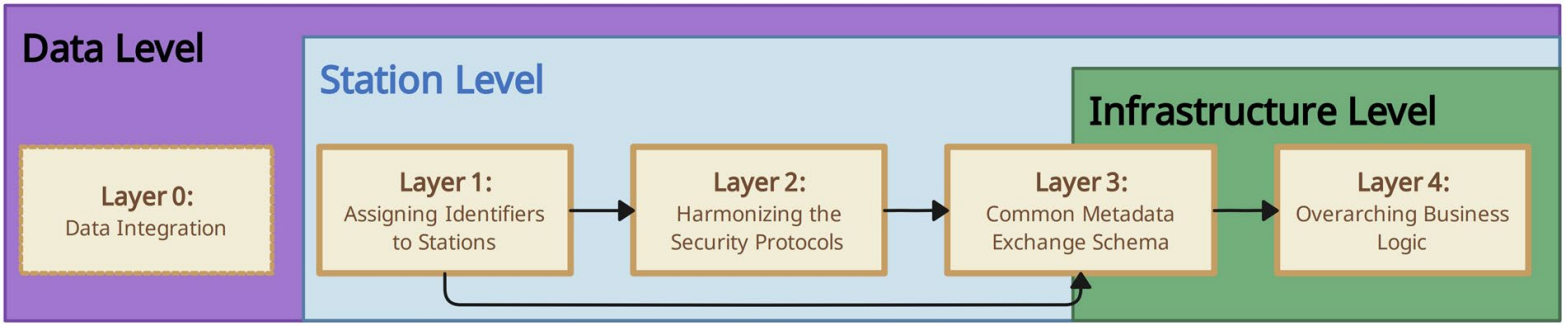
Our multi-layered framework for interoperability: From data integration to business logic. In our interoperability framework, Layer 0 is associated with the data level. The harmonization of station types, such as PADME or PHT-meDIC, is addressed in Layers 1 and 2, and to some extent in Layer 3. The overarching business logic is encapsulated within Layer 4 at the infrastructure level. The arrows illustrate the interdependencies and collaborative interactions across the layers: Layer 4 utilizes the metadata established by Layer 3 to navigate the trains through the infrastructures. Layer 3 consolidates the metadata produced by Layers 2 and 1. Layer 2 then uses the unique station identifiers to secure the trains accordingly.

**Fig. 3:**
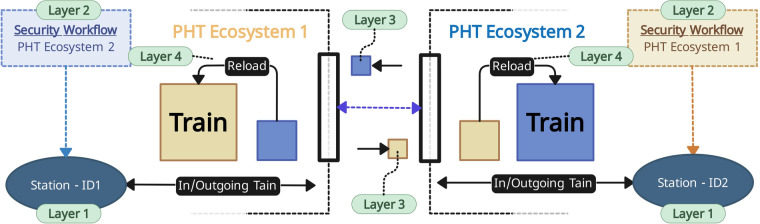
Our concept for the train transfer between PADME and PHT-meDIC. Each ecosystem has a dedicated interface that can receive trains. After the train of another ecosystem arrived, it is ‘reloaded’ into an ecosystem-specific and -compatible train (Layer 4). This can be interpreted as a ‘transfer station’ in the real-world, where cargo is reloaded from one train to another. Since each ecosystem adheres to its specific security protocol and the reloaded train contents are encrypted according to this protocol, we modularized each security protocol and make it available for the other ecosystem and vice versa. This is then used at the stations in order to decrypt (Layer 2) the contents - see Fig. 4 - using the metadata (Layer 3) attached to the train. The stations can be accessed through their unique station identifier (Layer 1).

Among our key outcomes was the formulation of five distinct layers to achieve PHT interoperability. This layered methodology draws inspiration from the ‘Layers of Interoperability’ by Benson *et al*.^19^. Our layers have been derived from the review in the *Methods* section and their relationship is visualized in Fig. 2 and Fig. 3. The five layers can be summarized as follows:

- **Layer 0 - Data integration**. The foundational step in our multi-layered approach involves harmonizing data across different infrastructures. This layer focuses on aligning and integrating the different data formats, structures, and standards from various data sources into a unified format such that it can be seamlessly processed by the analysis train.
- **Layer 1 - Assigning (globally unique) identifiers to stations**. In order to transfer trains between infrastructures, it is necessary to establish a method for identifying the station unambiguously across infrastructural borders. This is essential to ensure the correct routing of trains between the infrastructures and stations.
- **Layer 2 - Harmonizing the security protocols**. The PHT infrastructures were developed with different requirements regarding the security protocols and the encryption of the train. Therefore, we formulate an overarching security protocol that aligns with infrastructure-specific requirements.
- **Layer 3 - Common metadata exchange schema**. By employing distinctive station identifiers (Layer 1), we establish the initial building block of a shared communication standard. As the security protocol also requires metadata for proper functioning (e.g., exchange of public keys), our third objective is to create a common set of metadata that facilitates technical interoperability and also extends to a first foundation for semantic compatibility. This layer primarily merges the metadata items from Layers 1 and 2 into a machine-readable format.
- **Layer 4 - Overarching business logic**. After we have established all the preliminaries mentioned above, we need to develop the actual business logic to transfer trains between the infrastructures from a technical perspective based on the route defined by the identifiers (Layer 1).

In the following, we describe each of the layers in more detail.

### Layer 0 - Data integration

#### Conceptualization

In our scenario, the challenge lies in integrating three decentralized datasets from different institutions. Each institution houses its raw data in various clinical application systems. For example, as mentioned above, the data provided by Tübingen and Leipzig is highly specialized with respect to both childhood and adult variants of leukodystrophies, while Aachen only provides data on differential diagnoses. This data is unstructured and varies significantly in volume, format, and content, posing a significant challenge for standardization and integration. Therefore, a central data repository is needed in each institution that consolidates this diverse data in a more structured and accessible format. A key aspect of this integration process is the definition and implementation of a mutual data schema, such as the German MII Core Data Set (https://www.medizininformatik-initiative.de/en/medical-informatics-initiatives-core-data-set). This schema provides a standardized structure for the data, ensuring consistency and facilitating interoperability among the different institutions at the data level. Be aware that the design of such an ETL (Extract, Transform, Load) pipeline can vary widely, influenced by factors such as the nature of the data, the chosen data schema, and the specific technologies for data provision. In the subsequent section, we will briefly outline our specific strategy to harmonize the distributed data on leukodystrophy.

#### PoC implementation

In order to harmonize the data and make it analysis-ready, we store the data in a Research Electronic Data Capture (REDCap - https://www.project-redcap.org/) database system. REDCap is essentially designed for data collection and management in research studies and clinical trials and, therefore, suitable for our purpose^31,32^. At this point, we acknowledge that other technologies like Fast Healthcare Interoperability Resources (FHIR) could also facilitate data provision. However, in our scenario, REDCap was pre-installed at all participating sites and the medical teams were already familiar with this tool. For these reasons, we opted to use REDCap for our PoC. Each participating institution received a pre-compiled REDCap questionnaire, establishing a data schema to collect, structure, and standardize patient data across all institutions. The process of filling out the questionnaires for each patient has been performed manually or by semi-automatic means by the clinical teams in each hospital. The results of the REDCap questionnaire, which we refer to as the dataset later, is divided into three distinct sections:

- **Baseline:** Master data that contains patient details such as sex, age, and diagnosis.
- **Examination:** Data that provides information regarding the patient’s health status. This includes, among others, attributes such as abnormalities in higher brain functions or loss of libido that are defined in the Human Phenotype Ontology (HPO)^33^. The answers to the questions within this section encompass a wide spectrum of response types, spanning from yes/no/unknown options to open text fields.
- **Genetics:** Data that refers to genetics and serves as a documentation of the results obtained from genetic testing. This section includes details such as the observation year, the specific affected gene, and other attributes related to genes, such as the classification of the American College of Medical Genetics (ACMG) or the underlying mutation in the nomenclature of the Human Genome Variation Society (HGVS)^34,35^.

While the baseline section is mandatory for each patient, the others are optional and can consist of multiple instances (i.e., multiple examinations). In the final step, after all patient data instances have been entered into the REDCap system at each DIC, the REDCap database has been made accessible to the station software located at the respective DIC (see Fig. 1).

After the establishment of the data provision for the stations Aachen, Leipzig, and Tübingen, we need to move a layer up to ensure that the stations are accessible and can pull the trains. This includes assigning unique identifiers to the stations to provide a clear and unambiguous destination for the analysis train.

### Layer 1 - Assigning (globally unique) identifiers to stations

#### Conceptualization

In this layer, we address the disambiguation of the stations within a PHT ecosystem, especially during the route selection process. Up until now, the infrastructures have been using their own custom station identifiers, creating a potential risk of ambiguities due to the absence of a shared agreement on these identifiers between different infrastructures. Furthermore, the lack of global identifiers prevents one from choosing stations that belong to a different ecosystem for the train route. Therefore, we employ an additional authority on top of the infrastructures that acts as an indexing or directory service. In general, this approach is inspired by the well-established Domain Name System (DNS), which manages the namespace of the Internet^36,37^. Establishing such a namespace for the stations is our main objective, such that we can select stations for the train route and support the routing through our business logic (see Layer 4). In essence, we require that each station’s identifier must be a Uniform Resource Locator (URL). This URL represents the destination where the train is to be directed, enabling its execution at that particular station.

#### PoC implementation

Taking inspiration from DNS servers, we use the Station Registry (SR - https://station-registry.de/) as a component that serves as a central database for stations. Note that the SR has already been partially introduced in the work by Welten *et al*.^38^. Station administrators must register their stations at the SR before or after the installation and specify the affiliation of the station, e.g., in our case, either as a PADME or a PHT-meDIC station. Similarly to DNS, we have chosen a basic hierarchical structure for our station identifiers:

$$\mathop{\underbrace{{\rm{https}}://{\rm{station}}\mbox{--}{\rm{registry}}.{\rm{de}}/}}\limits_{{\rm{authority}}}\mathop{\underbrace{{\rm{PADME}}/}}\limits_{{\rm{affiliation}}}\mathop{\underbrace{{\rm{d7b0a9a7}}\mbox{--}07{\rm{fd}}\mbox{--}{\rm{4a31}}\mbox{--}{\rm{b93a}}\mbox{--}{\rm{3c946dc82667}}}}\limits_{{\rm{UUID}}}$$

Using these URLs, the business logic (as described in Layer 4) can effectively resolve the URLs and orchestrate the routing of trains between stations. This process is analogous to how requests are navigated on the Internet, starting from a higher level (the PHT ecosystem) and moving down to a more granular level (the individual stations). This hierarchical structure allows us to precisely locate each station, determine its association with a particular ecosystem, and dispatch the trains accordingly.

After establishing a method to identify each station through its URL, our next step involves synchronizing the internal workflows of each station type, whether it is a PADME or PHT-meDIC station. In our study, this specifically entails the alignment of the security protocols used by each station, which is part of the next section.

### Layer 2 - Harmonizing the security protocols

#### Conceptualization

**Fig. 4:**
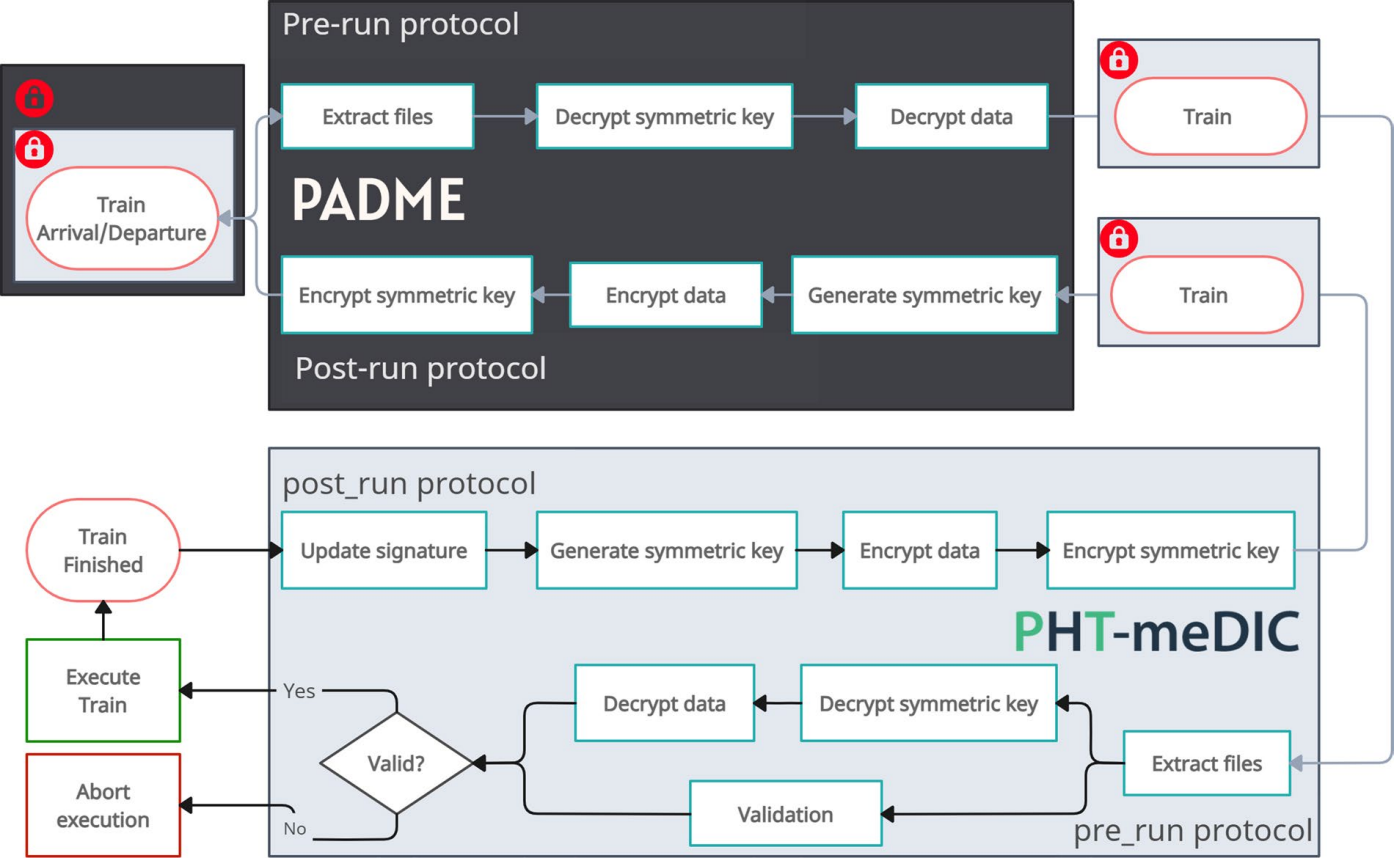
Interaction of the security concepts of the PADME and PHT-meDIC platforms. The overarching security concept for each infrastructure represents a combination of both. In our scenario, the PADME security protocol is applied on top of the PHT-meDIC protocol, when the train is at a PADME station. At a PHT-meDIC station, the order of the protocols would be flipped: first the PHT-meDIC, then the PADME protocol.

The next aspect that we need to address is the conceptualization of an overarching security concept, coupled with the alignment of the PADME and PHT-meDIC security protocols. One crucial challenge is that each infrastructure adheres to its own protocols and workflows with respect to encryption or the signing of digital assets. To overcome this, we propose adopting modular software containers, a methodology suggested by Hasselbring *et al*.^24^, to enhance software and workflow portability. Consequently, our strategy involves the containerization of all security-related processes of each ecosystem’s workflows to enable interoperability^24^. With this strategy, we achieve the flexibility required to implement these workflows across various ecosystems, while also ensuring the seamless distribution of protocol updates without substantial modifications in other ecosystems. The extracted containerized workflow can serve as a supplemental service, accessible for other PHT ecosystems, allowing different infrastructures to integrate this service into their own workflows. Alternatively, a comparable and probably less resource-intensive approach to distributing security protocols in a container could involve structuring them in a library-like structure that is installed in each involved infrastructure. All in all, the overarching protocol requires the containerized process steps to be arranged in sequence (see Fig. 4 as an example). The train undergoes decryption (pre-run) or encryption (post-run) incrementally, with each containerized module being invoked in succession to perform the necessary operations. The practice of nesting various workflows has the additional advantage that it can be applied multiple times, especially when more than two infrastructures are involved in the process. Having this concept in mind, we argue that the concept of this overarching security protocol, potentially comprising several sub-protocols, adheres to the established security policies of each PHT infrastructure involved, due to the sequential execution of each sub-protocol. In the following, we provide one exemplary integration of a security protocol in the context of our PoC.

#### PoC implementation

In our PoC, the security and encryption protocols of the PHT-meDIC ecosystem have been modularized through containerization. The container code can be found in the supplementary materials. This module is then seamlessly integrated into the execution environment of the PADME station(s). Note that this modular approach is reciprocal; the PADME protocol can also be similarly containerized and integrated within the PHT-meDIC station. Conceptually and metaphorically speaking, we consider this module image as a supplementary train that can be pulled and placed next to the *real* train that arrives at a station. During train execution, both security protocols are executed in sequence while preserving the integrity of the original system’s processes. The operation of this series of security protocols, as exemplified by our two protocols, is shown in Fig. 4 and works as follows:

- **Train arrival (Beginning of process):** Upon arrival at a PADME station, the train was secured with dual encryption: The external layer conforms to the PADME protocol, whereas the internal layer is encrypted following the PHT-meDIC protocol.
- **Pre-run protocol of PADME (Decryption - Outer layer):** The initial step involves the PADME pre-run protocol decrypting the external encryption layer.
- **Pre-run protocol of PHT-meDIC (Decryption - Inner layer):** After the decryption of the outer layer, the PHT-meDIC pre-run protocol decrypts the inner encryption layer, preparing the train for its execution.
- **Train execution:** Upon successful validation, the train is executed by the station environment.
- **Post-run protocol of PHT-meDIC (Re-encryption - Inner layer):** Post-execution, the train is re-encrypted, starting with the PHT-meDIC post-run protocol addressing the inner layer.
- **Post-run protocol of PADME (Re-encryption - Outer layer):** Following the inner layer’s re-encryption, the PADME post-run protocol re-encrypts the external layer.
- **Train departure (End of process):** This marks the completion of the entire train lifecycle at a station. After this step, the train is dispatched to the next station on the route.

In Layers 1 and 2 described above, we implicitly required metadata for each dispatched train. This includes details such as the station’s URL for precise train routing and security-related items such as the public keys of each station for encryption, which are especially crucial in Layer 2. To integrate these pieces of information into a single entity, we propose the creation of a unified data exchange schema. This schema will be instrumental in the decryption and encryption processes of the stations, as well as in directing the train on a given route. The formulation of this schema will be discussed below.

### Layer 3 - Common metadata exchange schema

#### Conceptualization

The implementation of a modular security protocol (Layer 2) also requires the presence of metadata (e.g., public keys, hashes, or signatures), which plays a crucial role in ensuring proper functionality. Consequently, based on the initial two layers outlined, we identify an additional requirement: the establishment of a standardized metadata schema. As already pointed out by Lamprecht *et al*., software metadata is a necessity for (semantic) interoperability^18^. This corresponds to the definition of Benson *et al*., which characterize semantic interoperability as the capability *‘for computers to share, understand, interpret and use data without ambiguity’*^19^. Hence, our aim is to develop a standardized set of metadata elements that can be used and exchanged in diverse workflows, ensuring that *‘both the sender and the recipient have data that means exactly the same thing’*^19^. We aim for a solution that can be effortlessly expanded to incorporate security-related and infrastructure-specific requirements. We divide the metadata set into two categories:

- **Business Logic Metadata:** This information is used by the business logic workflow, enabling tasks such as the routing of trains between stations. Within Layer 1, we have initiated the disambiguation of the stations by globally identifying them and incorporating them into the business logic metadata.
- **Security Protocol Metadata:** This category contains essential elements of modern security protocols and (asymmetric) encryption systems, such as public keys, hashes, and signatures that enable secure communication and data transmission by ensuring confidentiality, integrity, and authenticity^39^. The information provided can be utilized by the infrastructures to either apply external security protocols (as described in Layer 2) or their own protocols.

At this point, the question of the location of metadata storage for subsequent processing arises. Given the various possible approaches, such as centralized or decentralized storage, we require that the set of metadata is attached to the train and that each infrastructure handles it for internal business logic. This aligns with the train definitions proposed by Bonino *et al*., where trains are decomposed into metadata and the payload that contains the analysis^27^. Attaching metadata to the train offers the benefit of eliminating the need to query this information from external services. This approach is also autonomous and does not rely on any central authority, which ensures that the information is readily available wherever it is required.

#### PoC implementation

**Fig. 5:**
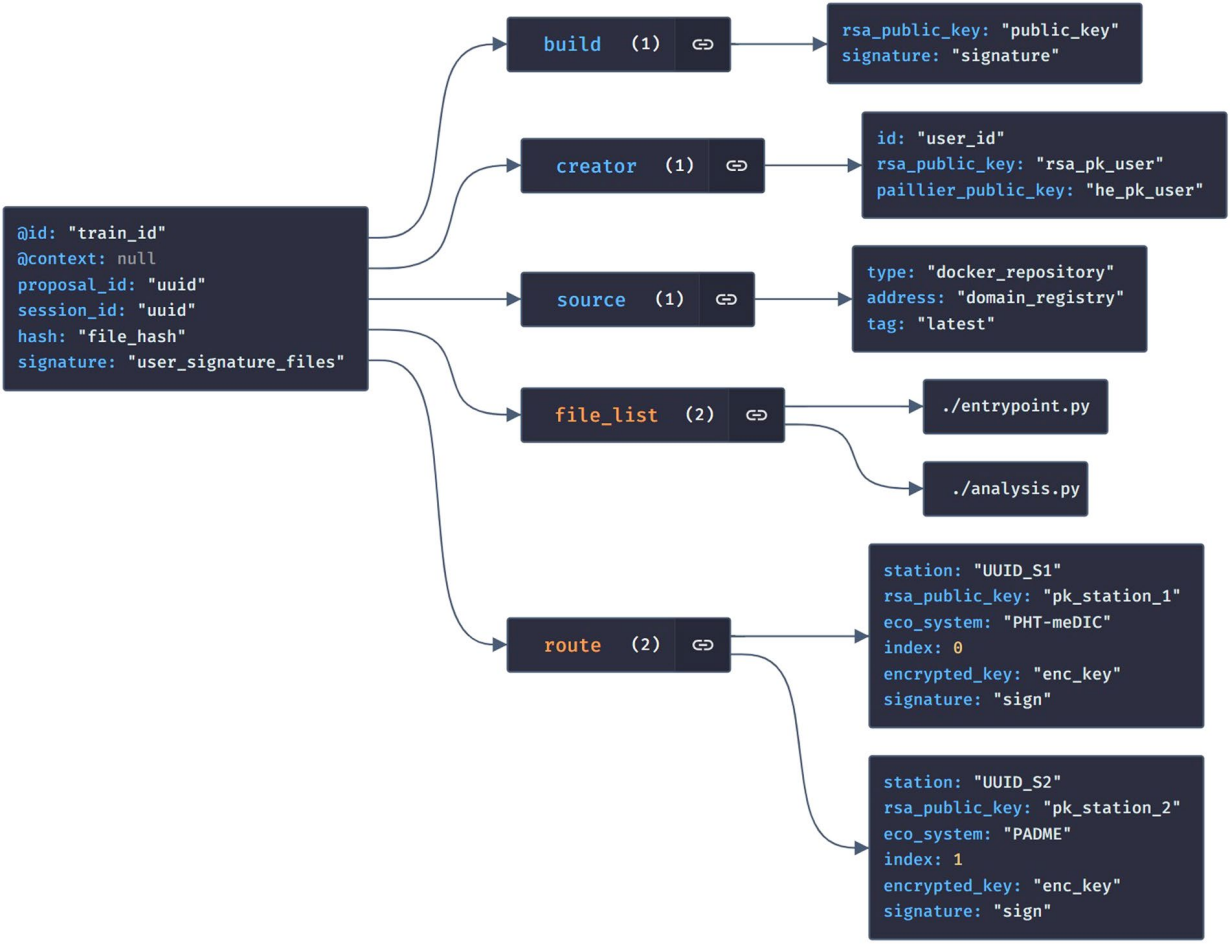
Metadata schema to enable interoperability. The schema contains information of two categories: Business Logic and Security Protocol metadata. Business Logic metadata is required for the train orchestration, while the security-related metadata is used by the security protocols.

According to our conceptualization, we use a small selection of metadata items as depicted in Fig. 5. This schema covers business-related information concerning the train’s origin (e.g., the source repository), its creator, and the identifiers of the stations to be visited. Note that these (basic) items share similarities with the definitions outlined by Bonino *et al*.^27^. Specifically, the route array captures essential station-related information and the station identifiers that have been introduced above in Layer 1. As each route item has a specific index the route can be systematically executed step by step. Additionally, the metadata includes the public keys of all participating entities, guaranteeing that assets can be encrypted in a manner that only the designated recipient can decrypt them. This is achieved in collaboration with the security protocols outlined in Layer 2, which process these keys. In terms of the integrity of the train contents, we also utilize signatures and hashes. In our particular case, each station’s public key is obtained from the SR. The installation and registration process of a station in the SR necessitates the provision of its public key, which is then retrieved from the SR during the route selection. Before the train is dispatched, the metadata is attached to the train.

Once we have established an identifier mechanism (see Layer 1), aligned the security protocols involved (see Layer 2), and laid the groundwork for a common metadata exchange schema (see Layer 3), the next step is to facilitate actual technical interoperability. This implies that we leverage the work established in the previous layers to facilitate the exchange of actual trains between the two ecosystems, PADME and PHT-meDIC. In the following section, we will introduce a routine that outlines the business logic required for this process.

### Layer 4 - Overarching business logic

#### Conceptualization

The objective of this layer is to perform the transfer of the analysis from one ecosystem to another. As the metadata attached to the train entails the concrete route information, the business logic is able to transfer the digital assets representing the train to a given destination. The destination endpoint can be a container repository, identified by a URL, to which the train can be sent for further processing. There might be several approaches and techniques to transfer and transform a train from one ecosystem to another. In the following, we briefly present our overarching business logic used to enable technical interoperability between PHT-meDIC and PADME.

#### PoC implementation

**Fig. 6:**
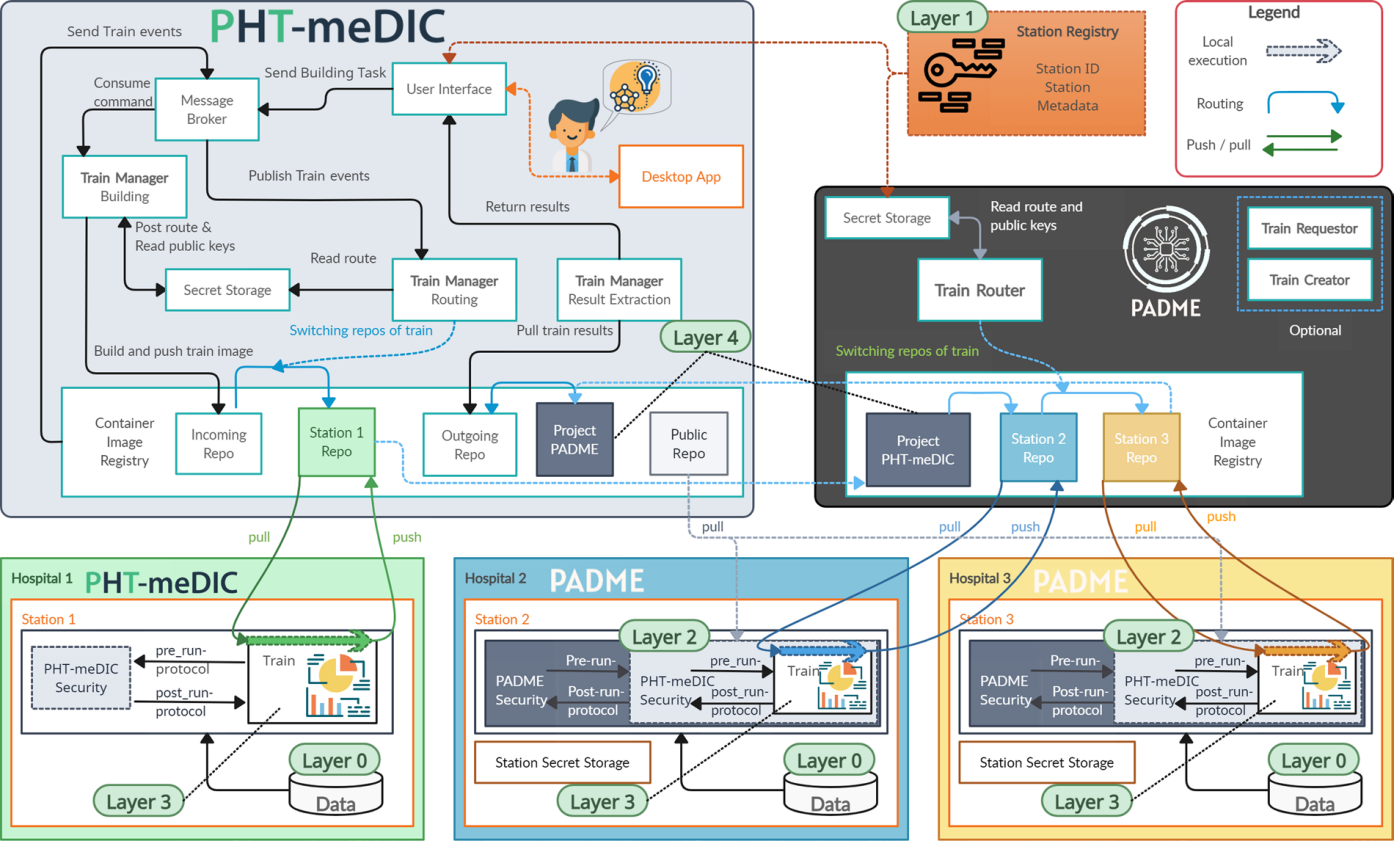
PoC implementation of our interoperability concept involving the platforms PADME and PHT-meDIC. This figure shows the routing of the train from PHT-meDIC to PADME. The reverse route, from PADME to PHT-meDIC, follows a similar pattern.

We refer to Fig. 3 and Fig. 6 as a graphical representation of our overarching business logic to transfer (container) trains between ecosystems. From a top-level perspective (see Fig. 3), our business logic is modeled after the real-world practice of reloading cargo from one train to another. The encrypted contents (the cargo) of the train, such as code and files, are unloaded from the arriving, external train and loaded into a new train that is compatible with the ecosystem.

From a technical perspective and to streamline the transfer of trains between ecosystems, we established a dedicated input repository (the ‘transfer station’) within each central service component. This repository is the designated location to which external trains should be pushed. A webhook monitors this repository, identifies incoming trains, and initiates the infrastructure-specific business logic to direct the train to its intended station after the train contents have been reloaded. This portal-inspired design decision has the advantage of keeping the actual stations private within the infrastructure, with only one repository accessible by external services or infrastructures. To send a train on its track, the entity requesting the train can select a route that utilizes information from the SR, potentially including stations within the same infrastructure, as well as those that connect different infrastructures. If the next station is assigned to the current infrastructure, the standard business logic proceeds to dispatch the train. In the other case, when the next station is assigned to another infrastructure, the business logic needs to proceed differently: As shown in Fig. 3 and Fig. 6, the business logic of the sending infrastructure detects the external station based on the metadata and transfers the train to the dedicated repository of the receiving infrastructure using a ‘push’ command (when using Container Trains). Subsequently, the receiving infrastructure’s business logic handles the train and prepares/reloads it for the next station along the route. The station receives the train and decrypts the envelope encryption using the modularized security protocol that has been introduced in Layer 2. After the execution of the train, it is re-encrypted using the envelope encryption and pushed back to the central service. Depending on the route, the train either remains in the infrastructure and is made available for the next station or is forwarded to the other infrastructure, following the same principle as described above. This cycle continues until all stations along the route have been processed.

Up to now, we have enabled the interoperability of the two involved ecosystems. Hereafter, we describe the evaluation of our PoC in the real-world setting of a research project.

### Conducting cross-infrastructural analysis

**Fig. 7:**
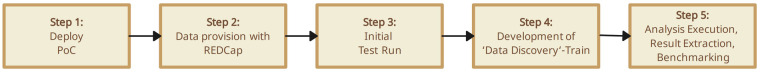
Validation process for the PoC. Step 1 encompasses the deployment of the PoC (Layer 1–4). In Step 2, we set up the data provision using REDCap. Step 3 involves an initial test run to ensure operational functionality. Step 4 is dedicated to the development of the ‘Data Discovery’ train that performs the analysis. The final Step 5 includes executing the analysis, extracting results, and benchmarking our PoC to acquire quantitative performance metrics for our interoperability concept.

Upon completion of our PoC development, we validate our approach within a real-world scenario and real patient data. For our interoperability study, we have opted to conduct a basic statistical analysis of the leukodystrophy data at hand. Since our concept is based on Container Trains, we argue that other trains with various analytical functions will also operate successfully within our PoC. The process of our evaluation is shown in Fig. 7.

After we have deployed our described components in the previous section and in Fig. 6 (Step 1), we set up a station at each of the three sites - Aachen, Tübingen, and Leipzig - and ensure that the REDCap databases at these locations are accessible via these stations (Step 2). After passing the functional tests (Step 3), our infrastructure is now operational. With access to the data, we can proceed with data analysis. The train design is part of the next section (Step 4).

#### Designing the ‘data discovery’-train

**Fig. 8:**
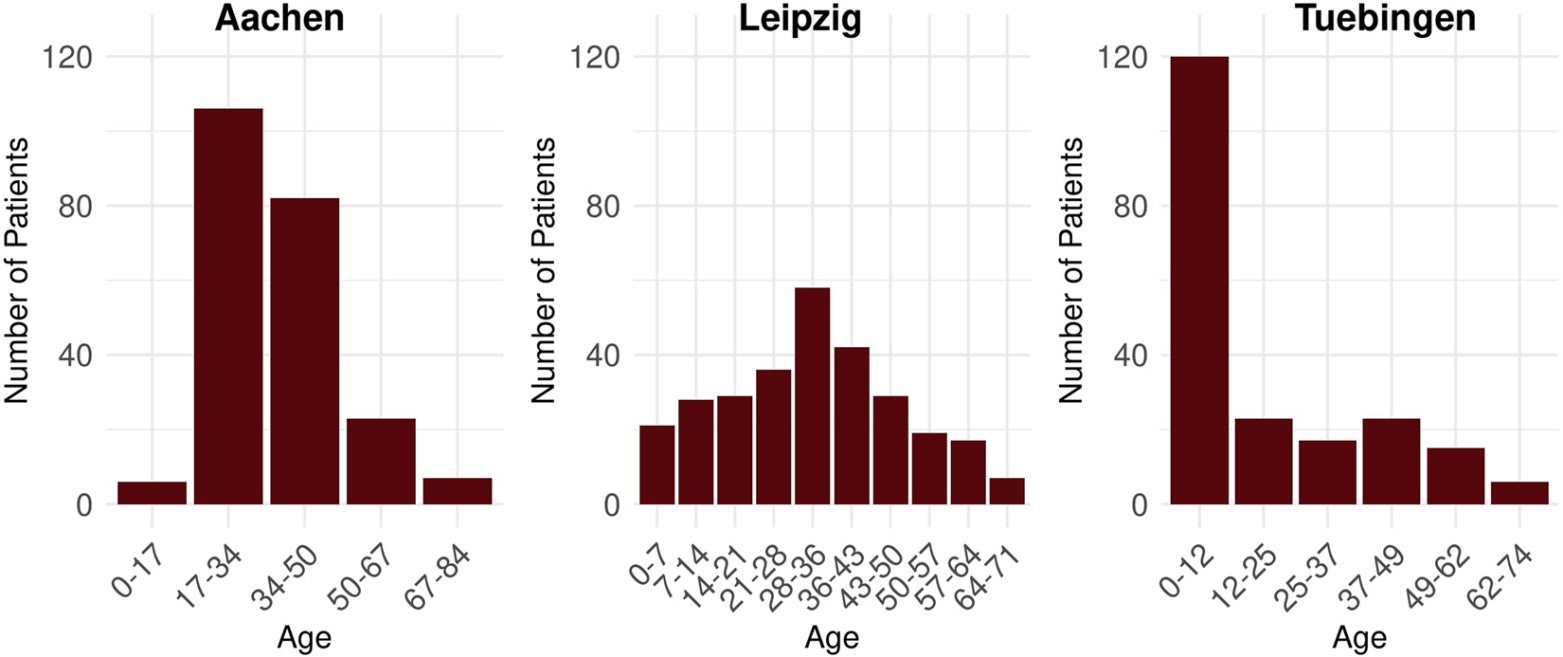
The age distribution from the Aachen, Leipzig and Tübingen. We organized the number of patients into bins that are tailored to ensure compliance with a k-anonymity threshold of k = 5^30^.

**Fig. 9:**
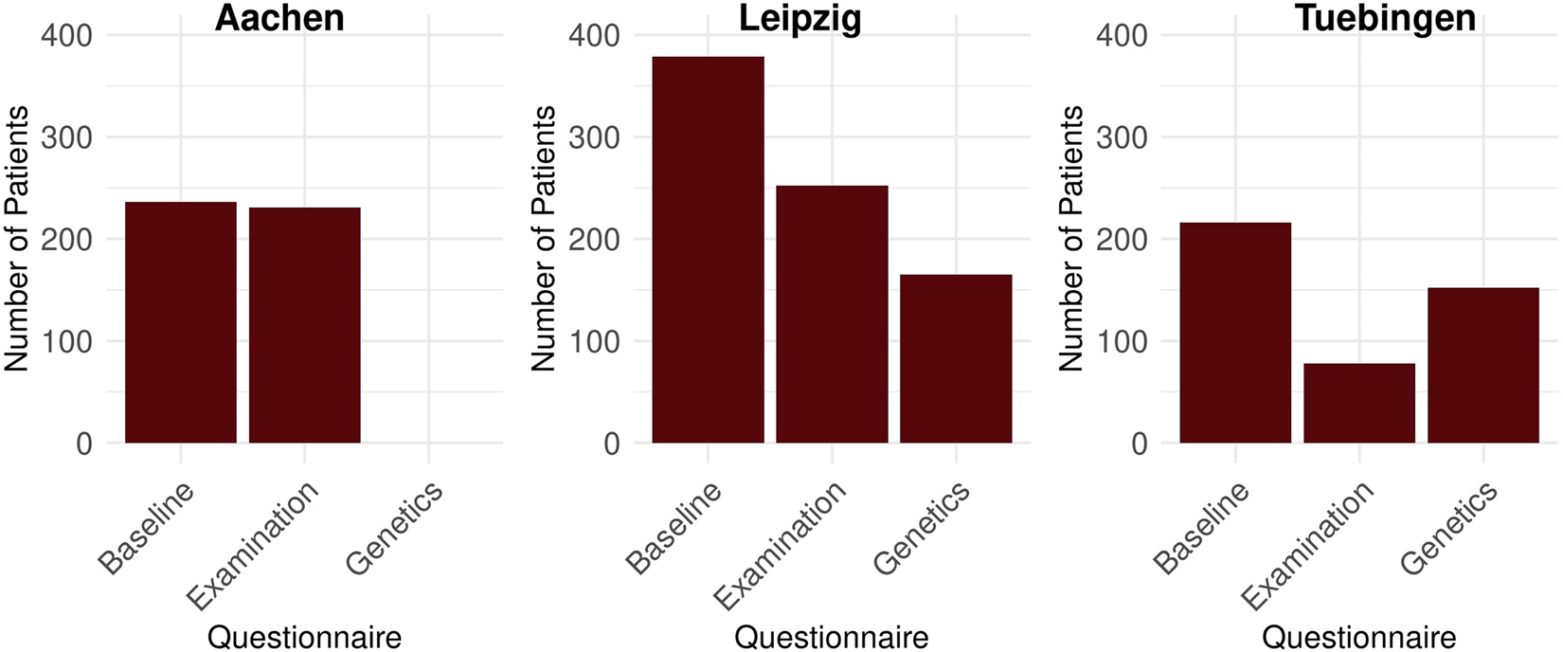
The count of patients across the three sections: Baseline, Examination and Genetics^30^.

The purpose of this study ‘Data Discovery’ is to initially inspect the data provided by the DICs, which, for example, can 1) support the identification of analogous studies that can be used as a reference point, 2) provide insights into the data quality, or 3) can be used as starting point for the design of a more sophisticated follow-up data analysis study. Since our work focuses on Container Trains, we create the train for our data analysis using Python (Version 3.10 - https://peps.python.org/pep-0619/) and Docker (https://www.docker.com) as containerization technology. The train consists of two steps. The initial step involves loading the REDCap data into the train, while the subsequent step involves the core data processing unit responsible for generating statistics and producing a PDF report for researchers. To streamline the data querying process, we have developed a custom data importer routine. This routine retrieves the data via direct access to the REDCap database, using the necessary credentials entered by the station administrator before initiating the train execution. For the creation of the report representing the analysis results, we gather several statistics. First, we determine the total number of male and female patients at each station (Table 1). Furthermore, we determine the age distribution at each station according to a k-anonymity level of 5 (Fig. 8)^40^. Lastly, we create plots showing the counts of the Baseline, Examination, and Genetic questionnaires (Fig. 9).

**Table 1:** Number of males and females from the stations in Aachen, Leipzig, and Tübingen^30^.

(a) Aachen	
Sex	Count
Male	140
Female	95

(b) Leipzig	
Sex	Count
Male	179
Female	71

(c) Tubingen	
Sex	Count
Male	116
Female	100

#### Execution and results of the analysis

After creating the Container Train, we executed the train (Step 5) according to our workflow depicted in Fig. 6. In summary, Figs. 8 and 9 provide an overview of the data underlying each station, providing information, such as the age distributions or the number of the Baseline, Examination, and Genetics sections in the questionnaire. The investigation of the age distribution, as shown in Fig. 8, indicates that in Tübingen, there is a balanced representation of male and female patients, most under the age of 20 years. This demographic trend is probably a result of the focus of the medical center on the treatment of leukodystrophies in children. On the contrary, Leipzig has a higher proportion of male patients and the majority of them are adults. Lastly, Aachen and Leipzig contribute more male patients than female (Table 1). Importantly, the dataset from Aachen is missing genetic data, as shown in Fig. 9, in contrast to the complete sets of questionnaires available from Leipzig and Tübingen. Measuring the impact of our interoperability concept in terms of the size of the dataset (number of patients), we come to the following conclusion. In cases where only one infrastructure is used, we either have 485 patients (Aachen + Leipzig) or 216 (Tübingen). However, through the interoperability of both ecosystems, we have achieved a total patient count of 701 (Aachen + Leipzig + Tübingen). These results highlight the success of our approach, enabling us to expand the total dataset and enhance our data-sharing capabilities.

## Discussion

In this section, we briefly discuss our results from the feasibility study, our interoperability approach, and the pros and cons of multi-homing compared to our approach.

### Feasibility study

Our data analysis within our feasibility study demonstrates the functionality of our interoperability concept (RQ1.1). We state that the execution of the ‘Discovery Train’ within the scope of our PoC implementation has been successful, demonstrating that our interoperability concept including global identifiers, harmonized security protocols, a common metadata exchange schema, and an overarching business logic enables technical interoperability between PHT infrastructures. Although our PoC implementation is functional, we encountered several challenges throughout the development of our train, primarily stemming from the distributed nature of both infrastructures. One significant challenge was related to the debugging of the train. Due to the slight variations in the dataset (inconsistencies during data acquisition) between Tübingen, Leipzig, and Aachen, we encountered some minor bugs during the execution of our train. These bugs emerged as critical single points of failure, requiring an additional development cycle to identify and fix them. Therefore, resolving these bugs proved to be a rather time-consuming task, demanding a significant level of communication and coordination between the three sites. The station administrators needed to inspect the error message output for potentially sensitive information and subsequently provide a secure version of the error report to the train developers. To mitigate the occurrence of errors during the execution of the train, we identified two crucial factors that come into play. First, harmonizing the datasets through, e.g., the usage of REDCap across all participating stations is vital. Second, testing the train with mock data that closely resembles real data can help identify and resolve potential issues before they arise in the real execution environment. As a result, we conclude that the seamless flow of interoperability can be disturbed by debugging procedures during the analysis development phase. However, we argue that this challenge may also manifest itself within a single PHT ecosystem, even when there is no interoperability required. Another method to support the debugging process could involve employing a second schema, similar to the data schema, specifically for logging output. This would ensure that only privacy-compliant log information is generated and utilized for debugging purposes.

In general, the results of our study highlight the importance of the FAIRification of health data, particularly in terms of data interoperability, which we refer to as Layer 0 in our conceptual framework. Previous research, such as the FAIR4HEALTH project, has demonstrated that approaches like FL (in our case, the PHT), can benefit from FAIR data^41^. These alternative methodologies on the data level, in contrast to our REDCap approach, are driven by FAIRification processes using, e.g., standards like HL7 FHIR or other technologies of the Semantic Web^41–43^. Overall, our research aligns with and complements the objectives and outcomes of existing interoperability initiatives - with a focus to a different aspect of interoperability^44–46^. For instance, CrowdHealth focuses on data integration and interoperability, featuring an analytical platform built on top of the the data layer^44^. InteropEHRate propose standardized healthcare data exchange formats and protocols and aims to achieve semantic and technical interoperability through HL7 FHIR profiles^46^. A comparable approach is conducted by the SmartCHANGE project, where also FL has been applied^45^. In contrast, our efforts are directed towards enhancing technical interoperability between two already established (PHT) infrastructures, avoiding the creation of a new infrastructure from scratch. Our work leverages the containerization technology, which ensures that the (PHT) infrastructures are compatible with emerging and established data standards (e.g., HL7 FHIR), thus supporting these existing works on data interoperability^44–47^.

### Interoperability: A detailed analysis

As we have pointed out earlier, the aspect of interoperability is complex, multifaceted, and its assessment can encompass various dimensions, spanning from governance to technical perspectives. Since we focused on the technical aspects of interoperability, we will examine our concept of interoperability in accordance with the recommendations of Hasselbring *et al*.^24^. These recommendations are structured and based on the FAIR principles for research software *‘to make research software FAIR and open’*^24^. Especially for interoperability, they have introduced four suggestions that offer qualitative assessment dimensions for evaluating our concept:

1. **Provision of proper interface definitions in modular software architectures**:
  Both PHT-meDIC and PADME, were initially designed with inherent modularity. This modularity allows the separation of different software components, making it easier to integrate and adapt them for various purposes. Second, the SR is also a separate module of our global infrastructure that can be queried through Application Programming Interfaces (APIs). In our combined infrastructure, APIs serve as the bridge between different software modules, facilitating smooth communication and metadata exchange, e.g., the transmission of the trains. The security container also has predefined interfaces to de-/encrypt the train (see supplementary materials). This enhances interoperability and promotes reusability or scalability. We also involve dedicated public repositories that can be used to transfer trains from one ecosystem to another through predefined interfaces of the containerization technology.
2. **Conformity to established software standards**:
  We follow established standards of the World Wide Web Consortium (W3C) and the Request for Comments (RFC) of the Internet Engineering Task Force (IETF) to ensure that our metadata is interoperable and readable by other systems. In our specific context, we have chosen JavaScript Object Notation (JSON) as a widely recognized data interchange format. In terms of security and encryption, our schema encompasses all essential components (public keys, signatures, hashes) required for the implementation of well-established encryption standards, for example, as outlined in RFC 8017^39,48^. Further, we adhere to common communication protocols, such as http(s), to exchange trains between the infrastructures.
  In addition, in Layer 1 we use identifiers, such as UUIDs (RFC 4122), to provide clear and unambiguous identification for stations within both ecosystems^49^. This identification system follows a hierarchical and DNS-inspired concept that has similarities to the well-established routing principles used within the Web.
3. **Usage of software virtualization techniques for portability**:
  Both PHT-meDIC and PADME, already utilize containerization as a means to encapsulate their components and dependencies. Hence, the infrastructures follow this practice of interoperability from the beginning. A new aspect that arises from our concept is the containerization of security protocols, aimed at achieving portability and, consequently, fostering interoperability among diverse security protocols by nesting them. Containerization of the security protocol also enhances the scalability of our interoperability approach, as it allows for the seamless integration of additional security protocols into the respective stations.
4. **Participation in artifact evaluation processes to evaluate interoperability:**

Artifact evaluation processes in the context of research software are designed to assess the quality and reusability of the software artifacts produced during research, in our case, the containerized security protocols. Up to now, we currently have an open issue regarding participation in such an artifact evaluation process, but we are committed to promoting interoperability through transparency and the provision of well-documented assets. All of our artifacts in this study are open source, which aligns with the FAIR principles by encouraging community collaboration and auditing processes.

### Interoperability versus multi-homing

An essential element of the discussion revolves around the comparison between the benefits of interoperability and what we term multi-homing - *‘the situation in which users tend to use several competing platform services in parallel’* as defined by the Directorate-General for Communications Networks of the European Commission^28^. In our specific PHT case, this implies that a single institution deploys at least two station software applications from at least two distinct PHT implementations and operates them concurrently. This scenario would make interoperability, as described in this work, redundant. Throughout our data analysis and execution of the train, we identified various factors that posed challenges to the multi-homing of both platforms. In the following, we discuss several disadvantages of multi-homing associated with hosting multiple platforms at a single location, in contrast to achieving interoperability, to answer RQ2:

- **Increased Complexity:** Multi-homing introduces an increased level of complexity. Institutional personnel must undergo training to proficiently manage and utilize two distinct systems, which requires significant investment in education and adaptation. In our specific situation, for instance, we would need to provide training to the personnel in Tübingen for operating the PADME station.
- **Governance Challenges:** Maintaining two infrastructures can entail governance hurdles. This involves obtaining approvals and ensuring compliance for both systems, which can be cumbersome and time-consuming. Further, the introduction of a potential redundancy might raise the question of whether a second platform is even necessary. This would prevent the institutional authorities from multi-homing, which would lead to a *quasi* data silo as the data is only accessible through a single platform. This approach would limit the access of data to a certain initiative, project, or geographic area. In our case, for instance, PADME is set up in Aachen and Leipzig, while PHT-meDIC is based in Tübingen (see Fig. 1). However, data analysis should not only be limited to accessible locations but should also encourage cooperation between scientists and data providers on an interregional or international scale. Thus, achieving interoperability among these platforms allows for data access and sharing across different regions or initiatives. In the particular scenario we encountered in this work, the interoperability framework we developed allowed for the integration of data from three distinct institutions into our study that have been affiliated with different initiatives (e.g., SMITH or DIFUTURE).
- **Security Risks:** Especially in a sensitive environment such as healthcare, multi-homing opens the door to additional security concerns (related to the governance aspect). With two ecosystem endpoints active, institutions face additional attack vectors that could potentially make them more vulnerable to security breaches and vulnerabilities. Trivially, achieving interoperability with other platforms necessitates just one endpoint.
- **Resource Consumption:** Running several PHT stations simultaneously requires more resources. This results in increased operational costs and may strain the institution’s IT infrastructure (related to the increased complexity aspect). Depending on where the software is deployed (e.g., in a cloud environment), an increased consumption of the resources might also impact the financial resources of an institution.
- **Data Integration:** Depending on the specifics of the infrastructures, data integration becomes a challenge. Data must potentially be integrated multiple times and made accessible through the PHT station application. Since data integration is inherently a time-intensive process, this stage would involve duplicating and storing data multiple times to ensure compatibility with multiple infrastructures (related to the aspect of resource consumption). Additionally, each *multi-home* may establish its own data schema and involve a unique data storage technology. When a clear schema mapping exists, this necessitates an extra step of transforming data from one schema format to another. If there is no defined schema mapping, this process becomes even more complex. In this work, we addressed this challenge through our Layer 0 defining a global data schema for the provided data to enable interoperability on the data level for both infrastructures.
- **Analysis Development Overhead:** When adopting a multi-homing approach, analysis development becomes a duplicate effort. In a heterogeneous landscape, where certain institutions use multiple PHT applications, while others do not, analyses may need to be developed separately for each system. Finally, merging results, either ad hoc or manually, adds an additional layer of complexity and time-consuming work for analysis developers. The debugging overhead we mentioned earlier, which we have encountered during our feasibility study, could potentially also have a negative impact on this aspect, as it can occur twice. Especially, this is a challenge that can be addressed through interoperability. With interoperability, the analysis code only needs to be developed once and can be effortlessly distributed across the PHT ecosystems.

Based on these aspects and the results of our feasibility study, we conclude that the multi-homing of similar infrastructures presents various drawbacks with respect to efficiency, security, and governance, making interoperability desirable. Particularly with regards to governance, achieving interoperability emerges as a necessary, and possibly the sole, solution in scenarios where the landscape is heterogeneous and multi-homing is not practiced - see our scenario of the feasibility study. Additionally, at this level of scale, it cannot be assumed that there will be only a single data analysis infrastructure that covers either a nationwide or international scope and would make interoperability unnecessary. Therefore, considering these factors, interoperability could be an appropriate strategy to address the aforementioned challenges. Ultimately and importantly, it should be acknowledged that multi-homing can still be a viable option, especially beneficial when multiple infrastructures that offer complementary features, such as diverse functionalities, are hosted together.

### The costs of our interoperability concept

This brings us to the question of what the expenses or overhead associated with our concept of interoperability are. A crucial aspect to consider is the incorporation of the containerization of the security protocol(s). This addition involves an extra layer in the local protocol and introduces possible time and resource demands, as the additional container must be pulled before the train execution. In our PoC, the security container has a size of 460.23 MB, which can be pulled once for future reuse. Additionally, the security container is also considerably smaller than our train used in the feasibility study: 1.22 GB. In contrast with the multi-homing scenario, we would have to install either a station of approximately 2.9 GB (PADME) or a size of 1.7 GB (PHT-meDIC). Hence, our concept is more efficient in resources. Since our interoperability concept encompasses the integration of multiple security protocols (in the form of an envelope), it results in an extended overall duration of the security protocol at the stations. In our specific situation and with our involved hardware, both decryption processes require 3 seconds each, resulting in a total decryption time of 6 seconds. Conversely, the encryption procedures take 8 seconds for the first step and an additional 15 seconds for the second step. The retrieval of the security container takes 6 seconds and only needs to be done once. Consequently, we argue that the added time required for our nested encryption approach is relatively small compared to the manual effort involved in managing two concurrent infrastructures during analysis. Regarding our business logic, Layer 4, the reloading/copying of the contents from one train to another takes less than 10 seconds. However, this reloading depends on the size of the contents and whether the images are already in the cache. If they are not, they need to be downloaded once. Furthermore, the introduction of an additional authority at the top level, in turn, requires potential administrative efforts regarding the creation and maintenance of standardized global identifiers. In our PoC, the SR is considered as the central registry for stations. As we have shown in our previous work, SR can be seamlessly integrated into the station installation workflow^38^. Therefore, we argue that administrative efforts are manageable.

### Limitations

Despite our successful establishment of technical interoperability between two distinct PHT infrastructures, our approach has certain limitations. These limitations are as follows.

#### Initial step in interoperability

As emphasized by Benson *et al*., achieving full interoperability (e.g., in healthcare) involves multiple levels, covering processes, clinical factors, governance, and regulatory dimensions^19^. Our work constitutes only one layer (i.e. the technology layer) among several to achieve complete interoperability among PHT infrastructures. For example, while our metadata schema (Layer 3) incorporates a core set of necessary items to serve two security protocols and business logics, additional items may be required to ensure, e.g., semantic interoperability with additional ecosystems beyond the scope of our study. Therefore, further investigations are required to facilitate interoperability from various points of view. Furthermore, as previously noted, it is essential to integrate data following a specific schema. The data schema (Layer 0) we developed was designed exclusively for our feasibility study and is not necessarily generically applicable or transferable to other data studies. Hence, our contribution is further limited in terms of data interoperability.

#### Technology scope

Our study focused on technical interoperability between two specific PHT infrastructures with a limited technology stack. The suitability and evaluation of our approach on a larger scale, involving multiple PHT ecosystems with other technologies, remains open. Further, our concept is centered around the utilization of Container Trains. However, as pointed out by Bonino *et al*., there are other types of trains that do not depend on containerization^27^. Therefore, it remains to be addressed in future work to achieve interoperability among various types of trains and the corresponding platforms.

#### Interoperability concept

Related to the limitations associated with the technology stack above, our findings are limited by our chosen method of approaching our objective. The development of our layered methodology was principally guided by an in-depth analysis of the differences in the technologies to build our interoperability concept. Our five layers for PHT interoperability might be one potential approach, while there may be other strategies to tackle the technical interoperability of the PHT.

#### Hierarchical identifier system

We designed our concept with a hierarchical identifier system inspired by the DNS, involving the SR. However, the introduction of a top-level authority could introduce a single point of failure that potentially disrupts the entire workflow. We have attempted to mitigate the first risk by implementing a cache for station identifiers. Alternatively, decentralized identifiers might offer a solution to comply with other identity management systems^50^.

These limitations highlight areas for potential future research and development to enhance the capabilities and scalability of our interoperability concept. Furthermore, reaching agreements across the entire community on selected aspects can enhance the progress of creating interoperable PHT platforms. Our five introduced layers may offer initial guidance or impulses in this regard, such as fostering a community-wide consensus regarding global station identifiers or a standardized PHT security protocol that can be applied to multiple platforms. This work serves as an initial study in the context of the German PrivateAim project (https://www.medizininformatik-initiative.de/de/privateaim-sichere-verteilte-auswertung-medizinischer-daten), which is dedicated to the establishment of a collaborative platform for DA known as Federated Learning and Analysis in Medicine (FLAME). Our efforts have laid the conceptual and technical foundation for the development of this collaborative platform. As future work, we intend to intensify the cooperation between the PADME and PHT-meDIC infrastructure. Our aim is to shift our focus from technical interoperability to data interoperability, with the ultimate goal of improving the efficiency of DA with the PHT. To achieve this, we plan to utilize both existing and upcoming reference works from established initiatives in Germany. One additional aspect of our approach is the reduction of redundancies via our interoperability concept, which reduces energy consumption. Analyzing the energy efficiency of our approach could be a focus for future research. Since interoperability and energy consumption have already been explored in the context of the Internet of Things, this existing knowledge could be leveraged to evaluate the energy efficiency of our solution in subsequent studies^51–53^.

## Methods

In this section, we briefly discuss the relation between interoperability and the FAIR principles. We then present the core components of the two involved infrastructures, their similarities, and differences as part of a technology review. Finally, we outline our assumptions and the corresponding design objectives we made prior to the concept.

### The ‘I’ in FAIR

Organizing and managing well-structured scientific data serves as the basis for the performance of knowledge discovery systems and data analysis^13,54^. Therefore, the management and techniques for generating digital artifacts from research studies are essential for discovery, retrievability, evaluation, and reuse. To endorse this concept, a set of core guidelines called *FAIR Data Principles* was propagated in 2016^54^:

- **Findable**: Make the given dataset findable by maintaining clear, explicit, and rich metadata with a globally unique and persistent identifier. Data and metadata should be properly registered and indexed to make them searchable in the future.
- **Accessible**: Metadata and data should be retrievable using any standard communication protocol. The protocol includes an authentication and authorization procedure and is open-source and universally implementable.
- **Interoperable**: Both data and metadata use qualified, formal, accessible, and shareable vocabularies for the representation of knowledge with suitable references to other metadata or data.
- **Reusable**: The presence of a clear and accessible data usage license with detailed provenance and domain-relevant community standards makes a given dataset and its metadata reusable.

**Fig. 10:**
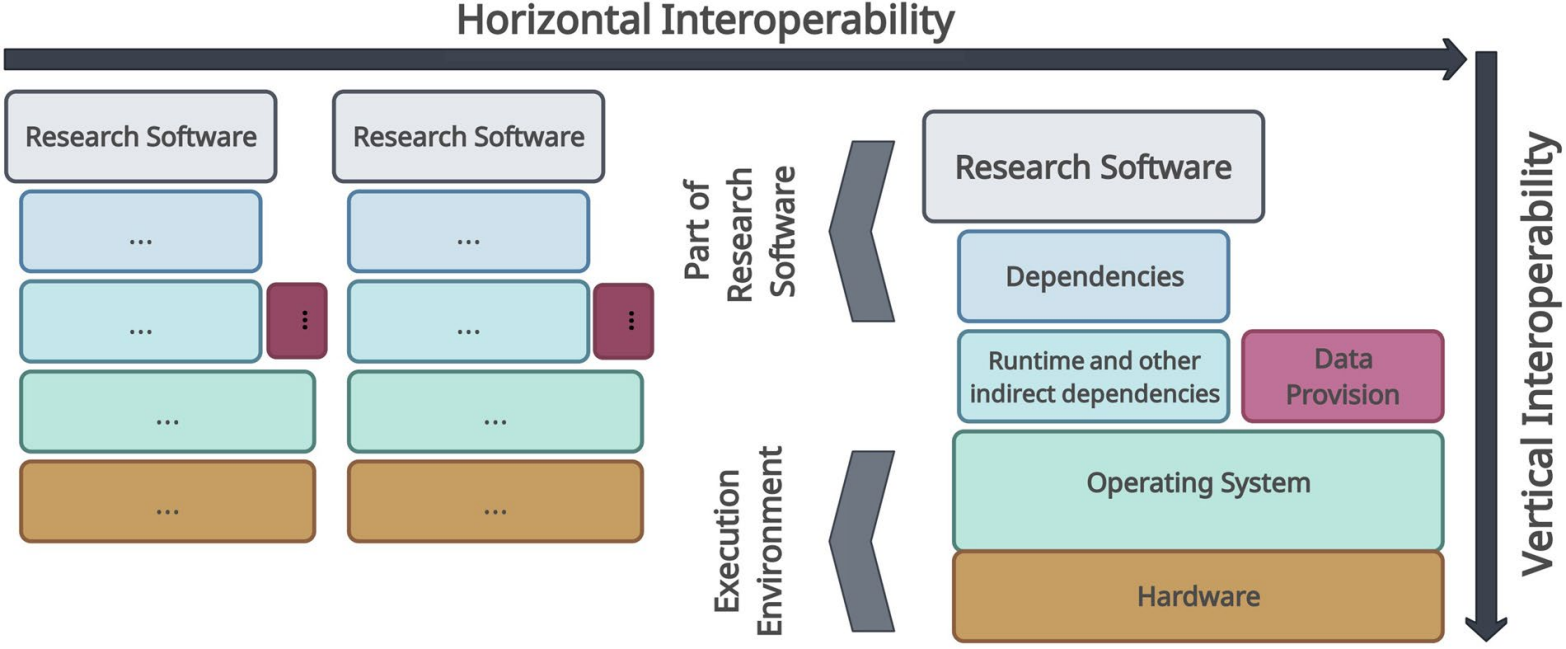
Refined definition of interoperability. Interoperability is divided into two main categories: vertical and horizontal interoperability. Vertical interoperability focuses on the compatibility of software objects within a stack, encompassing, for example, hardware-level drivers and executable programs. Horizontal interoperability pertains to the compatibility of multiple software stacks within a workflow. In this work, we consider a workflow as the execution of a train within a PHT infrastructure. Our objective is to achieve horizontal interoperability between two PHT infrastructures by connecting two of such stacks/workflows including the data provision. Adapted from Lamprecht *et al*.^18^.

Originally, the idea was that the FAIR principles apply to data and other assets such as research software, including workflows, tools, algorithms, and even software systems used in data processing^55^. Other studies or reports, such as the six recommendations for the Implementation of FAIR Practice by the FAIR in Practice Task Force of the European Open Science Cloud (EOSC) or the FAIR Principles for Research Software (FAIR4RS Principles) by the FAIR for Research Software Working Group (FAIR4RS WG), endorsed the need for FAIR research software to enhance reproducibility, transparency, interoperability and reusability of research^18,21–23,56,57^. As Lamprecht *et al*. pointed out, software per se cannot be considered as data^18^. Therefore, Lamprecht *et al*. refined the original definitions of the FAIR principles so that they can be applied to software^18^. Of interest for this work is the interoperability component of the FAIR principles. According to Lamprecht *et al*. and the IEEE Standard Glossary of Software Engineering Terminology, interoperability is the ‘*ability of two or more systems or components to exchange information and use the information that has been exchanged*’^18,19,58^. As this definition has kept the actual meaning of system or component generic, Lamprecht *et al*. introduced two dimensions of interoperability to address the variability in software assets (see Fig. 10)^18^.

According to the more refined definition of interoperability, we can distinguish between *horizontal* and *vertical* interoperability. Vertical interoperability defines the compatibility of a stack of software objects among each other, such as software close to the hardware (e.g., drivers) and executable programs on top of it (e.g., scripts)^18^. In the scenario of this work, vertical interoperability is achieved through the inherently incorporated paradigm of containerization, i.e., virtualization, in the PHT. Horizontal interoperability defines the compatibility of multiple software stacks as part of a workflow. In this work, we interpret such a single workflow as the execution of a train within a single PHT infrastructure. Therefore, based on this definition, our goal is to achieve technical horizontal interoperability between two PHT infrastructures, specifically, two software stacks.

### The concept of the Personal Health Train

As part of the well-known GO FAIR initiative, the PHT has been initiated as an enabler for the FAIRification of research data^18,55,59^. The fundamental concept behind PHT involves the practice of DA, which brings the analysis code to where the data resides. As the PHT approach plays an important role in this work, we provide an overview of the various components of the infrastructure. The PHT, as its name might suggest, draws an analogy from real-world railway systems and the trains that operate on them. The PHT comprises three core components: Train, Station, and Track (Handler)^9,13,27^. The Train created by a data consumer, e.g., a researcher, represents a data analysis. The purpose of the Train is to transfer the analysis between each data-holding institution, called Station, using the Tracks that connect these institutions. The Station, on the other hand, is an institution that provides and grants access to sensitive and confidential data that is processed by the Train. Each Station in the PHT infrastructure receives a Train, executes the analysis, appends the result to the Train, and passes the Train onto the next Station. As emphasized by Bonino *et al*., there may be various types of Trains, each serving different purposes and using different underlying technologies^27^. For example, a Train could be a Query Train, Script Train, or Container Train. The latter is relevant to this work. A Container Train encapsulates the algorithm integrated into an analysis program, along with all essential dependencies (libraries) and the execution environment, within an image or container, for example, using Docker. So far, the concept of the PHT has already shown its capabilities in several use cases, such as radiomics, hypertension, or lung cancer analysis^13,60,61^. Several implementations following the PHT concept have been developed up to now, such as vantage6 (https://distributedlearning.ai/), PADME, or PHT-meDIC^16,17,62^. Since PADME and PHT-meDIC are relevant for this work and our PoC, we discuss their implementations in more detail.

### Reference architectures

**Fig. 11:**
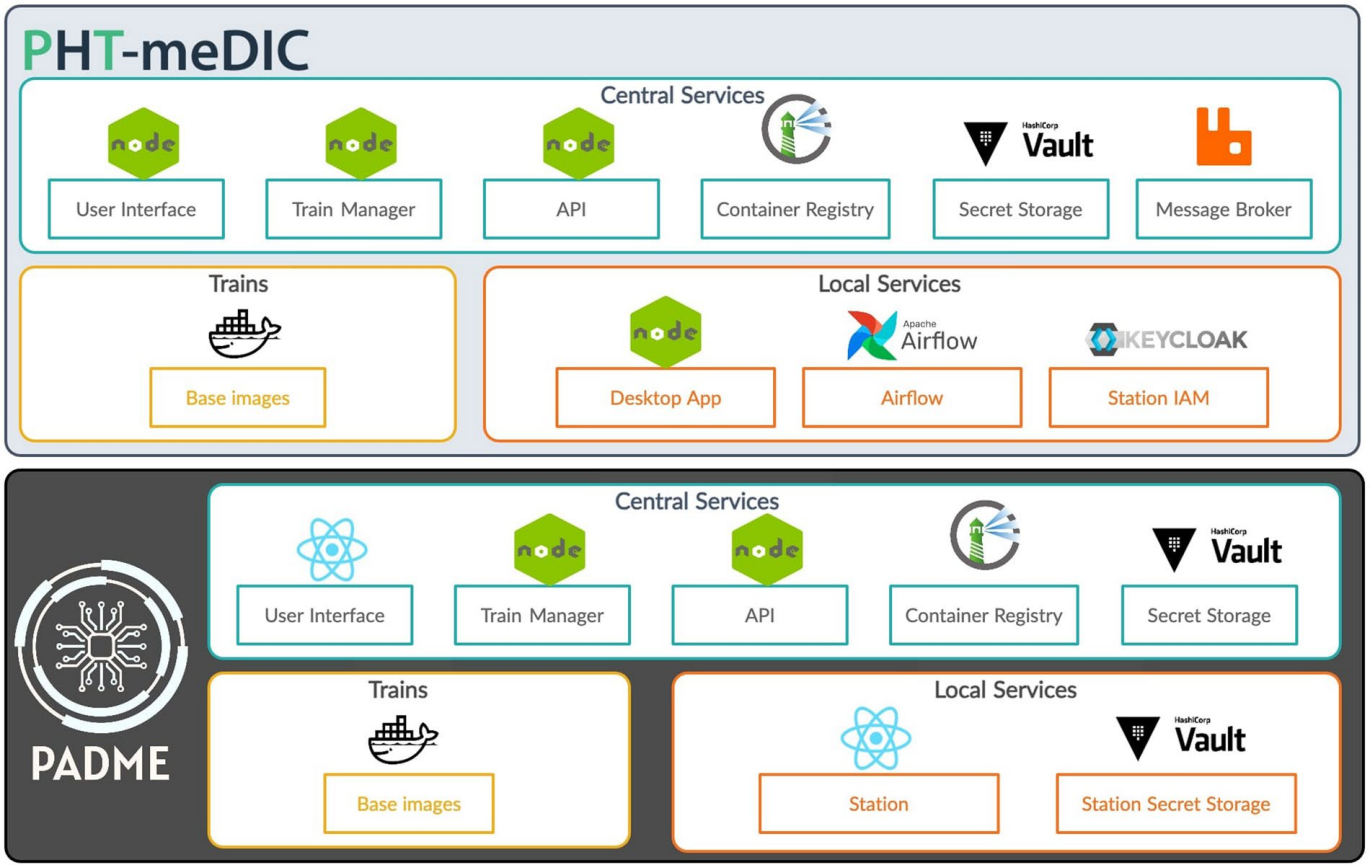
Overview of PHT infrastructures: PADME and PHT-meDIC. PHT-meDIC and PADME follow a container-based security-by-design approach, employing tools like Harbor and Vault for secure operations. Both consist of two primary components: the Central Service (CS) and the station software, also referred to as local service (LS). The CS manages train repositories, including the central train repository using Harbor. The station software operates as a control interface for the container engine. Encryption in PADME uses envelope encryption with symmetric and asymmetric keys. The security concept of PHT-meDIC also focuses on the detection of encryption and manipulation detection using digital signatures.

In this section, we briefly review the key components of each infrastructure and further indicate if and how the derived commonalities or differences support or complicate our plan for technical interoperability. For an overview, Fig. 11 provides a top-level perspective of the components and Table 2 gives a summary. For a detailed description of the infrastructures, we refer to the corresponding publications^16,17^.

**Table 2:** Comparison of aspects in the PADME & PHT-meDIC architectures and the relation to interoperability.

Aspect	PADME	PHT-meDIC	Interoperability
**Overview & Architecture**	Composed of Central Service (CS) for train orchestration and station software for train execution. Follows a star topology.	Composed of a central orchestration component and local service (LS) components. A central user interface for analysis requests. Follows a star topology.	Simplified due to consistent architectural design.
**Technology Stack**	Uses containerization with Harbor and Vault for train management and encryption. Custom web service for station software.	Uses containerization with Harbor and Vault for security. Employs Airflow and RabbitMQ for station services.	Established communication interfaces through containerization. Shared reliance on containerization. Challenges in aligning precise implementation details.
**Data Provision**	Freely selectable. There is no limitation to a specific technology.	Freely selectable. There is no limitation to a specific technology.	Given the variety of possible approaches, there is a need for a standardized data schema and individual data storage systems across each data-holding institution. Challenges in data integration necessitate a unified, interoperable data schema for analysis.
**Security Concepts**	Envelope encryption with symmetric and public/private key pairs. A public key is stored in the Vault system of the CS. Unique permanent key pairs for each station.	‘pre_run’ and ‘post_run’ security steps, envelope encryption, digital signatures for tamper detection, and unique pseudo-identifiers (UPIDs).	Comparable security workflows, including asymmetric encryption. Challenges in aligning implementation details. Integrity checks PHT-meDIC necessitate the employment of globally unique identifiers.

#### Overview and architecture

PADME, developed by Welten *et al*., is composed of two primary components for managing trains and data analysis^17^. The first component, the Central Service (CS), handles the reception and orchestration of trains following predefined routes. The second component, the station software or local service (LS), serves as the endpoint for executing these trains. Similarly, PHT-meDIC consists of two architectural components: a central orchestration component and LS components. Furthermore, the PHT-meDIC central user interface (UI) offers users the capability to request, approve, and submit analyses, with the added feature of providing a link to download the encrypted analysis results. The LS component also includes a desktop app, allowing users to manage key pairs, sign trains, and locally decrypt results. ***In terms of interoperability: Both infrastructures follow a star topology in their architectural design. This aspect facilitates and streamlines horizontal interoperability because there is no need to modify the internal architectural design of both infrastructures****.*

#### Technology stack

Both, PADME and PHT-meDIC, implement containerization for their components, creating fully containerized web applications. In PADME, the CS maintains a repository for each participating station, with a central train repository known as Harbor (https://goharbor.io). Meanwhile, the station software in PADME uses a custom web service to manage train arrivals and departures, which essentially acts as a remote control for the underlying container engine. The encryption and decryption of trains are handled by an encryption engine called Vault (https://www.vaultproject.io), which also locally manages the private key generated during the station installation. Similarly, PHT-meDIC is container-based and places a strong emphasis on security-by-design. It also utilizes Harbor and Vault to protect sensitive information during train orchestration. For long-running tasks, PHT-meDIC uses a message broker to distribute them asynchronously to microservices instead of processing them directly through APIs. Upon task completion, an API aggregates the results, updates the database records, and communicates the results to the user in real-time through a web sockets service. Unlike PADME, PHT-meDIC uses Airflow (https://airflow.apache.org) individually at each study site as a station, and it also uses the message broker RabbitMQ (https://www.rabbitmq.com) to facilitate communication between central services. ***In terms of interoperability: The technology stack of both infrastructures is largely similar, if not entirely compatible. The primary benefit of these infrastructures lies in their mutual dependency on containerization. This inherent feature establishes predefined and standardized communication interfaces, which can be leveraged for our interoperability concept****.*

#### Data provision

From the technology stack presented, it becomes evident that PADME and PHT-meDIC do not rely on a specific data provision technology. Each station software is designed to interface with any data storage technology, thereby facilitating access to the stored data for analysis purposes. ***In terms of interoperability: While the flexibility provided by both infrastructures in data provisioning is advantageous, it also poses difficulties in data integration as multiple approaches are conceivable. Therefore, a solution is required to establish a cross-infrastructural data schema and a data storage system at each data-holding institution****.*

#### Security concepts

Both, PADME and PHT-meDIC, implement security measures for the management of train-related data and the protection of sensitive information. In PADME, when a developer dispatches a train, it is moved to the dedicated repository of the respective station and only the station can pull the train. PADME relies on envelope encryption with symmetric and public/private key pairs for each train execution. The CS and each station have their unique permanent key pair. The encryption method changes based on the location of the train. It is encrypted with the public key of the subsequent station when the train is at the CS, and with the CS’s public key when it is at a regular station. In contrast, PHT-meDIC introduced a security concept with ‘pre_run’ and ‘post_run’ steps in 2019 (for additional information, see the supplementary materials). This concept serves three crucial purposes. First, it allows for quick encryption and decryption of results using envelope encryption to ensure data security at rest and in transit. Second, a chain of digital signatures is used to detect any tampering with analysis algorithms, data queries, and results at any stage. This enables individual stations to execute or abort analyses. Third, PHT-meDIC employs unique pseudo-identifiers (UPIDs) to safeguard against inference attacks or manipulation of the route definition. ***In terms of interoperability: Both infrastructures share comparable security workflows at the conceptual level. Both utilize asymmetric encryption, which can be advantageous for our interoperability concept. However, aligning the precise implementation details of these security workflows poses challenges. While the encryption methods used in both infrastructures are similar, PHT-meDIC also conducts integrity checks on the route definition, involving a list of station identifiers that both infrastructures must be able to access. Moreover, to start the encryption process, we need a solution that ensures that all encryption-related assets, including public keys, hashes, or signatures, are readily available for their respective security workflows****.*

Based on this review of both involved infrastructures, we formulated our five-layer interoperability concept to enable interoperability between these PHT infrastructures. ‘Layer 0 - Data integration’ refers to the phase of harmonizing data across the two infrastructures. We term it ‘Layer 0’ as it is more closely associated with the data-holding institutions, rather than the infrastructures themselves, which are agnostic to the data source. This layer becomes unnecessary if the involved institutions are already aligned with a common standard that ensures data harmonization. The necessity of ‘Layer 1 - Assigning (globally unique) identifiers to stations’ stems from the different station identification mechanisms used by both infrastructures. As we have pointed out above, both infrastructures diverge in their approaches to security protocols. Based on this fact, we have derived ‘Layer 2 - Harmonizing the security protocols’ to develop an overarching security concept. Given that ‘Layer 2’ relies on security-related metadata, ‘Layer 3 - Establishment of a common metadata exchange schema’ becomes essential. ‘Layer 3’ ensures the proper operation of the security protocol and the train routing by providing the necessary metadata in a standardized format. Ultimately, ‘Layer 4 - Unified business logic’ is required to orchestrate the transfer of a train from one infrastructure to another, leveraging the groundwork laid by ‘Layers 1’ through ‘Layer 3’.

### Assumptions and design principles

Our concept is based on various assumptions and guiding design principles that shape our concept. Mainly, we strive to maintain the existing infrastructures along with their inherent architectural designs. In other words, our goal is to ensure that the workflows, policies, and (security) protocols of both infrastructures remain largely consistent in their current state-of-the-art. Another benefit of this approach is that it does not affect the usability of the infrastructures or necessitate modifications to the local station, thereby keeping the interaction with the local software consistent. According to that, we follow the design principle of loose coupling between these infrastructures, primarily to accomplish two objectives: First, even when interoperability is not required, both infrastructures should remain independently operational, and the interdependencies should be kept to a necessary minimum. Second, this will enhance the adaptability of our concept for future interoperability with other PHT platforms. As mentioned by Hasselbring *et al*., essential for research software to be interoperable are modular software components that use well-defined interfaces^24^. Hence, we pursue a module-based approach through software virtualization and web services, i.e., containerization. These techniques contribute to enhanced portability, which itself fosters interoperability and the potential for reuse across additional (DA) platforms, beyond our two study-related infrastructures^24^. Similarly to loosely coupled components, we also want to leverage an asynchronous and event-driven approach to further contribute to the independence and autonomy of the infrastructures. Regarding the communication itself, we will rely on a standardized metadata exchange format and a common set of metadata that will comply with best practices and well-established standards of the Web.

## Data Availability

The data for Figs. 8, 9, and Table 1 can be found on Zenodo: 10.5281/zenodo.11101321^30^.

## Code availability

Code for the PADME platform: https://git.rwth-aachen.de/groups/padme-development. Code for the PHT-meDIC platform: https://github.com/PHT-meDIC. The implementation of the ‘Discovery Train’ can be found here: 10.5281/zenodo.11101321. The security container can be found: https://github.com/PHT-Medic/train-container-library and its explanation: https://difuture.de/wp-content/uploads/2019/10/Marius-Herr_DIFUTURE_Symposium_exported_High_quality.pdf.
